# HAlign-II: efficient ultra-large multiple sequence alignment and phylogenetic tree reconstruction with distributed and parallel computing

**DOI:** 10.1186/s13015-017-0116-x

**Published:** 2017-09-29

**Authors:** Shixiang Wan, Quan Zou

**Affiliations:** 1 School of Computer Science and Technology, Tianjin University, Tianjin, China; 2 Guangdong Province Key Laboratory of Popular High Performance Computers, Shenzhen University, Shenzhen, China

**Keywords:** Multiple sequence alignment, Phylogenetic trees, Distributed computing, Spark

## Abstract

**Background:**

Multiple sequence alignment (MSA) plays a key role in biological sequence analyses, especially in phylogenetic tree construction. Extreme increase in next-generation sequencing results in shortage of efficient ultra-large biological sequence alignment approaches for coping with different sequence types.

**Methods:**

Distributed and parallel computing represents a crucial technique for accelerating ultra-large (e.g. files more than 1 GB) sequence analyses. Based on HAlign and Spark distributed computing system, we implement a highly cost-efficient and time-efficient HAlign-II tool to address ultra-large multiple biological sequence alignment and phylogenetic tree construction.

**Results:**

The experiments in the DNA and protein large scale data sets, which are more than 1GB files, showed that HAlign II could save time and space. It outperformed the current software tools. HAlign-II can efficiently carry out MSA and construct phylogenetic trees with ultra-large numbers of biological sequences. HAlign-II shows extremely high memory efficiency and scales well with increases in computing resource.

**Conclusions:**

THAlign-II provides a user-friendly web server based on our distributed computing infrastructure. HAlign-II with open-source codes and datasets was established at http://lab.malab.cn/soft/halign.

## Background

Multiple sequence alignment (MSA) is a necessary step for analyzing biological sequence structures and functions, phylogenetic inferences, and other basic fields in bioinformatics [1]. Given the rapid increment of biological sequences in next-generation sequencing [2], difficulties arise from insufficiency of available state-of-the-art methods for addressing ultra-large sources.

Increasingly more different parallelization strategies are implemented for reducing time and space complexity of MSA. These strategies can be mainly categorized into three levels: multiple threads based on central processing unit (CPU) on a single machine, multiple threads based on graphics processing unit (GPU) on a single machine, and multiple threads based on CPUs or GPUs on cluster machines. CPU-based multiple threads, which are common and effortless, suit small-scale sequence alignment. With emergence of bottlenecks in increasing clock frequency of multi-core CPUs, Moore’s law became meaningless [3]. Based on NVIDIA GPU, compute unified device architecture (CUDA) technique was designed for efficient parallelism [4, 5]. GPU functions in real-time rendering of screens, because hundreds of cores in GPUs can efficiently calculate pixels or coordinates in parallel. However, under limited video memory size and bandwidth, alignment of ultra-large sequences becomes difficult or even impossible [6]. With high computational cost, most naive algorithms attempted to reduce time and space complexity to cope with ultra-large analysis tasks.

Recently, large-scale distributed computing was applied extensively to various biological analyses, such as ClustalW-MPI [7], Hadoop-BAM [8], HAlign [9], and HPTree [10]. For next-generation sequencing, CloudDOE [11], BioPig [12], and SeqPig [13] were implemented; these software benefited from using open-source distributed frameworks. Different from traditional single machine systems, distributed computing systems perform load-balancing for fault-tolerant parallelized tasks and can be easily extended to cheaper devices for improvement of computing power. Additionally, distributed computing systems based on MapReduce framework present more abstract interfaces and more elastic computing resources than those based on message passing interface (MPI) [14]. Ultra-large biological sequence analysis can be efficiently addressed by assembling distributed and parallel computing systems with numerous cheap devices [15–17].

Although HAlign software, which is based on Hadoop framework [18], exhibits better computing power and expansibility than other strategies running on a single machine. Apache Spark framework works up to 100 times faster than Hadoop, especially in iterative operators. Apache Spark can also accelerate real-world data analytics approximately 40 times faster than Hadoop and can even be employed to scan one TB data in five- to seven-second latency [19]. Based on Spark framework [20], Marek et al. developed SparkSeq [21], which can be used to analyze nucleotide sequences with considerable scalability. Zhao et al. developed SparkSW [3], which can carry out Smith–Waterman algorithm [22] in load-balancing way on a distributed system to cope with increasing sizes of biological sequence databases. However, SparkSeq can only work with nucleotide sequences but not with protein sequences; thus, Smith–Waterman algorithm in SparkSW cannot achieve peer performance on nucleotide sequences. Additionally, both SparkSeq and SparkSW are fairly suitable for developers, they do not support generation of phylogenetic trees.

We implemented HAlign-II based on HAlign work, HPTree work, and Apache Spark framework to address ultra-large multiple biological sequence alignment and construct large-scale phylogenetic trees. HAlign-II shows high memory efficiency with large-scale MSA and phylogenetic trees construction, scales well with increasing computing resources, and provides a user-friendly web server deployed on our infrastructure.

The rest of this paper is organized as follows. In the following section, we first introduce the Apache Spark framework. Based on Spark framework, respectively, we describe Smith–Waterman algorithm for protein sequence alignment, trie trees algorithm for nucleotide sequence alignment, and neighbor-joining (NJ) method [23] for phylogenetic trees construction. Thereafter, we present datasets and comparative experiments with state-of-the-art tools and evaluate memory efficiency and scalability of HAlign-II. Last, preceding experimental results are discussed, and conclusion of the study is provided.

## Methods

### Overview of Apache Spark

Apache Hadoop and Apache Spark are famous open-source frameworks in the field of distributed computing. Hadoop mainly contains Hadoop Distributed File System (HDFS) [18] for distributed storage and MapReduce programming model for big datasets [24]. HDFS stores data on inexpensive machines, providing dependable fault-tolerant mechanism and high-aggregate bandwidth across clusters. Spark aims to blueprint a programming model that extends applications of MapReduce model and achieves high computational efficiency-based memory cache.

**Fig. 1:**
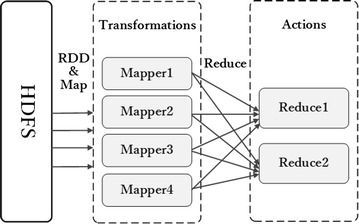
A simple Spark workflow

Spark designs an abstract data structure named resilient distributed datasets (RDDs) [19] to support efficient computing and to ensure distribution of datasets on cluster machines. RDDs support extensive variety of iterative algorithms, a highly efficient SQL engine Shark, and a large-scale graph computing engine GraphX. RDDs staying in memory cache will visibly reduce load time when requiring replication, especially in iterative operations. From Fig. 1, to further reduce time and cost, two types of operations in RDDs are designed: transforms and actions [19]. Transforms only deliver computing graphs, which only describe how to compute and not how to carry out computing operations, such as map and filter operation. Actions carry out computing, such as reduce and collect operations, results of which are stored as new RDDs. Based on these operations, RDDs are efficiently executed in parallel. To ensure dependable fault tolerance, RDDs will be recomputed after data loss, for example, because of halting of individual machines. Based on RDDs, Spark can implement up to 100 times theoretical speed than Hadoop in real-world datasets [19].

### Smith–Waterman algorithm for protein sequences with Spark

With its high sensitivity, Smith–Waterman algorithm [23] can locally align object and subject sequences to obtain similarity segments based on dynamic programming; however, global alignment results cannot be obtained. In the past decades, this algorithm was cited over 8000 times in the biological field.

Smith–Waterman algorithm can search the best alignment location through given scoring methods, such as substitution matrix and gap-scoring scheme. Negative scoring matrix cells of this algorithm are set to zero, which is necessary for achieving alignment location. Traceback procedure of alignment starts from highest scoring matrix cell and proceeds until a cell with score of zero is encountered, thereby yielding the highest local alignment scoring. Suppose that *n* and *m* correspond to respective lengths of *A* and *B* sequences, then substitution matrix and gap-scoring scheme are respectively represented by $s\left( {a, b} \right)$ and $W_{k}$. Then, Smith–Waterman algorithm creates scoring matrix H and initializes the first row and column; the process can be formulated as follows:

1 $$H_{k0} = H_{0l} = 0, \left( {0 \le k \le n, \quad 0 \le l \le m} \right).$$

Then, the rest of matrix H should be filled with similarity scores, which are formulated as follows:

2 $$H_{ij} = max\left\{ {\begin{array}{l} {\begin{array}{l} {H_{i - 1,j - 1} + s\left( {a_{i} , b_{j} } \right),} \\ {max_{k \ge 1} \left\{ {H_{i - k,j} - W_{k} } \right\},} \\ \end{array} } \\ {\begin{array}{l} {max_{l \ge 1} \left\{ {H_{i,j - l} - W_{l} } \right\},} \\ 0 \\ \end{array} } \\ \end{array} } \right. \left( {1 \le i \le n, \;1 \le j \le m} \right).$$

where $H_{i - 1,j - 1} + s\left( {a_{i} , b_{j} } \right)$ represents similarity scores between *a*_*i*_ and *b*_*j*_, *H*_*i*−*k*,*j*_ − *W*_*k*_ corresponds to matched scores when *a*_*i*_ points to the end of a *k* length gap, *H*_*i*,*j*−*l*_ − *W*_*l*_ is the matched scores when *b*_*j*_ points to the end of a *l* length gap, and 0 indicates absence of similarity.

**Fig. 2:**
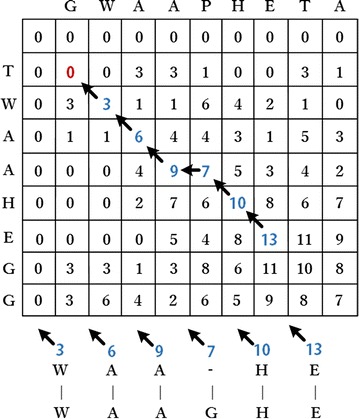
Traceback procedure and pairwise alignment results of Smith–Waterman algorithm

Figure 2 shows gradual traceback from the highest-score matrix cell to lowest-score matrix cell, looping to dynamic programming based on zero-score matrix cell. The algorithm obtains inserted space positions and generates pairwise alignment results.

As high time and space complexity of Smith–Waterman algorithm poses challenges concerning ultra-large datasets, this paper implements this algorithm on distributed computing system based on Spark framework.

**Fig. 3:**
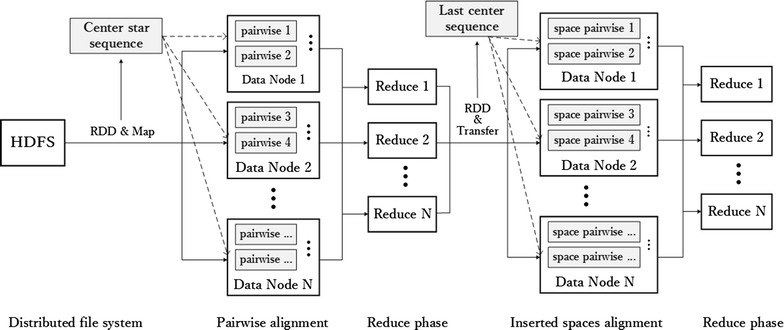
MSA procedures based on Spark distributed framework

As shown in Fig. 3, the entire processing procedure is partitioned into two MapReduce steps. In the first step, the extracted center star sequence based on Smith–Waterman algorithm becomes a broadcast variable to align other sequences for filling inserted space matrix cells; this sequence records positions and numbers of inserted space. Then, first reduction generates the last and longest center star sequence for further calculations. Score matrix and center star sequence are cached in memory, spreading the center star sequence to each data node. Next, final pairwise alignment is initiated by inserted space matrix and each individual sequence. Finally, HDFS stores MSA results.

### Trie trees method for similar nucleotide sequences with Spark

Smith–Waterman algorithm is accurate and mature and thus is suitable for protein sequence alignment of complex structures and elements. However, to obtain high similarity of most nucleotide sequences during alignment, running time of Smith–Waterman algorithm extremely increases, especially with ultra-large nucleotide sequences. Hence, this work considers tree-based data structures to address the problem in ultra-large nucleotide sequence alignment. A series of MSA methods about tree-based data structures are applied; such methods include BLAT [25] and Hobbes [26]. According to HAlign [9], trie tree serves as an efficient data structure for storing multiple sequences; this structure quickly indexes common sub-strings from long strings and accelerates MSA search. A trie tree only features one root node and *n* leafs for *n* nucleotide sequences [27]. Additionally, trie tree can speed up search in linear running time by failure links.

Two primary steps can be used to realize MSA based on trie tree: select a center star sequence for pairwise alignment and to integrate inserted spaces. Center star sequence contains the most segments among all sequences, thereby implying that it is the most similar to other sequences. As large-scale nucleotide sequences are similar, the first sequence represents the center sequence. Thereafter, other sequences are aligned to center sequence based on unmatched segments from the trie tree. In HAlign-II, this step is designed as numerous highly parallel operations across data construction of RDDs and is partitioned into memory on multiple workers. Pairwise alignment costs linear running time instead of exponential running time. Suppose that *n* similar nucleotide sequences with average length of *m* exists. Then, time complexity of trie tree algorithm is *O*(*n* ^2^ *m*); trie tree algorithm requires less running time than the original center star method (time complexity is *O*(*n* ^2^ *m* ^2^)). For *n* − 1 times pairwise sequence alignment, time complexity is $O\left( {nm^{2} } \right)$. However, practical time consumed is far less than theoretical value because matched segments are skipped in high sequences. If $n \ll m$, then practical time consumed can be regarded as linear. In the last step, multiple alignment results are partitioned into new RDDs and delivered to multiple distributed workers for calculation. Center star sequence and its alignment results spread to entire Spark cluster as shared similar constants, as presented in Fig. 3, to further reduce running time.

### NJ method for constructing phylogenetic trees with Spark

Frequently, MSA is required before constructing phylogenetic trees, such as MAFFT, MEGA, IQ-TREE, FastTree, iGTP, SATe-II, phangorn and our NJ method. However, most MSA tools cannot address large or ultra-large numbers of sequences. Based on MSA and Spark framework, this paper implement NJ method for constructing phylogenetic trees.

Phylogenetic trees can be built using distance-based, maximum parsimony, and maximum likelihood approaches [10]. NJ approach [23] represents one of the distance-based approaches, and according to HPTree work, it is time-efficient and suitable for ultra-large sequences data.

**Fig. 4:**
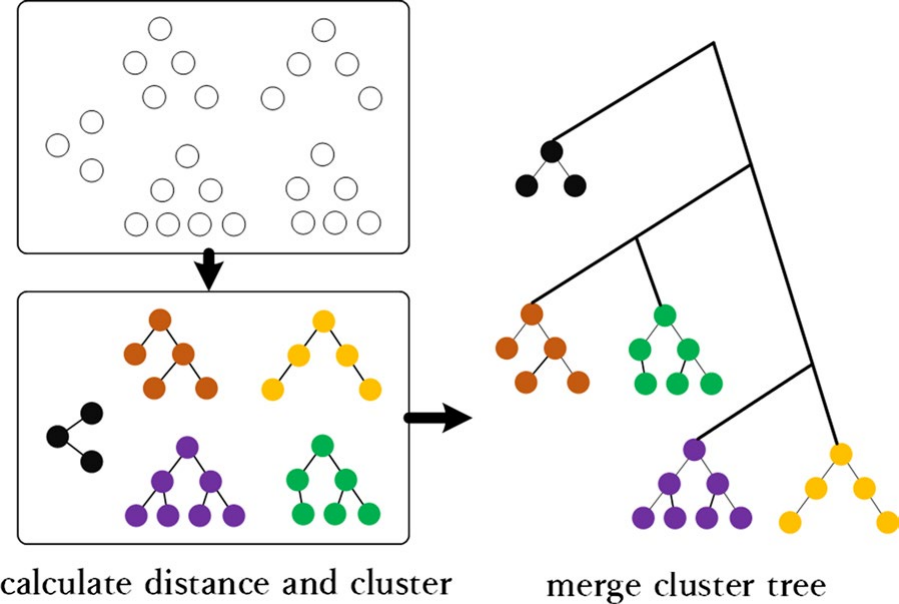
Constructing phylogenetic trees based on distance measure

As shown in Fig. 4, based on parallel computing, we first cluster all MSA results into several clusters. Then, we calculate individual phylogenetic tree based on individual clusters. Last, all phylogenetic trees are merged on clusters into the final evolution tree. We highlight the initial clustering procedure. Approximately 10% (a changeable threshold value) of all MSA sequences are selected by random sampling for initial clustering. Then, functional distance of each pairwise MSA sequence is calculated, clustered, and labeled until all sequences are identified. When few clusters whose number of elements is over 10%, then they are merged into other clusters; otherwise, they are divided into more balanced clusters until balanced construction. The entire procedure is designed for Spark parallel model in Fig. 4.

## Results and experiments

### Datasets and metrics

The main research object of HAlign-II is ultra-large biological sequences dataset included protein sequences and nucleotide sequences. For protein sequences datasets, BAliBASE [28] is regarded as golden benchmark, with BAliBASE 4 as the newest version. The tests in the BAliBASE 4 benchmark suite are divided into 10 different reference sets. For each test, a number of files are provided, while all files derived from different families contain the sequences corresponding to the homologous regions only. And a family in a reference set is matched to a given reference alignment for evaluating. Each family need to be aligned simultaneously. In order to focus on the performance with increasing sequences scale, we employ the newest and largest R10 reference set Φ_*Protein*_ as our protein sequence datasets (that is, 218 MSAs are independently constructed).

According to previous HAlign work [9], we use human mitochondrial genomes Φ_*DNA*_ and 16s rRNA Φ_*RNA*_ as nucleotide sequences datasets [29]. After MSA, phylogenetic trees are generated by MSA results on Spark platform. Table 1 shows more detailed information regarding biological datasets.

**Table 1:** Original dataset and datasets after threshold removal

Dataset	Number	Minimum length	Maximum length	Average length	File size
Φ_*DNA*_(1 ×)	672	16,556	16,579	16,569.7	10 MB
Φ_*DNA*_(100 ×)	67,200	As above	As above	As above	1.1 GB
Φ_*DNA*_(1000 ×)	672,000	As above	As above	As above	11 GB
Φ_*RNA*_(small)	108,453	807	1599	1442.8	156 MB
Φ_*RNA*_(large)	1,011,621	807	1629	1388.5	1.4 GB
Φ_*Protein*_(1 ×)	17,892 (218 families)	19	4895	459.0	15 MB
Φ_*Protein*_(100 ×)	1,789,200 (218 families)	As above	As above	As above	1.5 GB
Φ_*Protein*_(1000 ×)	17,892,000 (218 families)	As above	As above	As above	15 GB

The original bali score program in BAliBASE used a different way of handling gaps in segments, which resulted in incorrect normalizations, so that even perfect multiple alignments could have a score less than one. To determine the similarity of the alignment obtained by a program to the reference alignment in BAliBASE, column score (CS) and sum-of-pair score (SPS) are calculated as two alignment scores according to Karplus’s work [30]. The CS counts the number of columns of the segments that are aligned correctly in all sequences, normalized by the number of alignment columns. It is noticeable however, that one badly misaligned sequence reduces CS from 1 to 0. Indeed we have observed that CS tends to be almost a binary value—with each alignment either being very good or scoring 0. The SPS counts how many pairs of residues are correctly aligned. Suppose that there is an alignment with N sequences of length of M, if in column *i*, both sequence *x* and sequence *y* have residues aligned in a segment of the reference alignment, then pair value *P*_*ixy*_ equals 2; if one of both alignments has a gap, then *P*_*ixy*_ equals 1, otherwise *P*_*ixy*_ equals 0. The total score is normalized by the maximum possible score, so that the range of possible values is from 0 to 1, with 1 indicating a multiple alignment that is identical on the segments. The score *S*_*i*_ with the *i*th column and SPS are

$$\left\{ {\begin{array}{l} {S_{i} = \sum\limits_{{j = 1}}^{N} {\sum\limits_{{k \ne j}} {P_{{ijk}} } } } \\ {SPS = {{\sum\limits_{{i = 1}}^{M} {S_{i} } } \mathord{\left/ {\vphantom {{\sum\limits_{{i = 1}}^{M} {S_{i} } } {\sum\limits_{{i = 1}}^{{M_{r} }} {S_{{ri}} } }}} \right. \kern-\nulldelimiterspace} {\sum\limits_{{i = 1}}^{{M_{r} }} {S_{{ri}} } }}} \\ \end{array} } \right..$$

where *M*_*r*_ is the number of columns in the segments of the reference alignment and *S*_*ri*_ is the score *S*_*i*_ for the *i*th column in the reference alignment. For achieving better evaluation of ultra-large-scale alignments, we employ average SPS as the final metric of MSA experiments.

In essence, the higher the SPS or is, the more accurate is the alignments generated by the programs. In this paper, we calculated the alignment scores for evaluating protein and nucleotide alignments.

As HAlign-II contains three types of biological sequence alignment and phylogenetic tree construction based on Spark platform, our experimental environment consists of a cluster comprising 12 workstations. Each workstation features 384 GB physical memory with Intel Xeon E5-2620 processors, and each processor contains eight processing cores. Based on Ubuntu 16.04 operating system and Spark 2.0.2, a series of experiments are presented in succeeding sections.

### Comparison with state-of-the-art tools

We select a series of state-of-the-art tools to compare with HAlign-II and evaluate its performance on addressing ultra-large datasets. Our comparison eliminates Kalign [31] is completely unsuitable for large-scale datasets. Similarly, phangorn [32], RAxML [33], Pasta [34], and STELLS [35] are eliminated because of their nearly intolerable time consumption. In particularly, Clustal Omega [36], designed for handling data-sets of hundreds of thousands of sequences in reasonable time, has no edge than other state-of-the-art tools in our large-scale datasets. As should be mentioned, SparkSW method uses Spark version 1.0; however, the newest version used in our cluster is 2.0, which performs better in theory. Additionally, we deploy the newest Hadoop framework on our cluster for running HAlign.

*Experiment (a)* Based on MUSCLE (version 3.8, fast parameter “− maxiters 2” is used, the same below) [37], MAFFT (version 7.3, fast parameter “− parttree” is used, the same below) [38], Clustal-Omega (version 1.2.4, default mode is used, the same below) [36], HAlign, and HAlign-II tools, we implement ultra-large multiple similar genome sequence alignments with Φ_*DNA*_(1 ×), Φ_*DNA*_(100 ×), and Φ_*DNA*_(1000 ×) datasets.

*Experiment (b)* Based on MUSCLE, MAFFT, Clustal-Omega, HAlign, and HAlign-II tools, we implement ultra-large multiple dissimilarity RNA sequence alignments with Φ_*RNA*_(small) and Φ_*RNA*_(large) datasets.

*Experiment (c)* Based on MUSCLE, MAFFT, Clustal-Omega, SparkSW, and HAlign-II tools, we implement ultra-large multiple dissimilarity protein sequence alignments with Φ_*Protein*_(1 ×), Φ_*Protein*_(100 ×), and Φ_*Protein*_(1000 ×) datasets. It is noticeable that MUSLE, MAFFT, SparkSW and HAlign-II align each family in the reference set, respectively.

*Experiment (d)* Based on IQ-TREE (version 1.5.5, multithread mode) [39], HPTree, and HAlign-II tools, we construct ultra-large phylogenetic trees with Φ_*DNA*_(1 ×), Φ_*DNA*_(100 ×), Φ_*DNA*_(1000 ×), Φ_*RNA*_(small), Φ_*RNA*_(large), Φ_*Protein*_(1 ×), Φ_*Protein*_(100 ×), and Φ_*Protein*_(1000 ×) datasets. For our HAlign-II method, we initially align multiple sequences and then build phylogenetic trees.

Tables 2, 3, and 4 respectively show all experiment results with genome MSA, RNA MSA, and protein MSA. Surprisingly, MUSCLE exhibits extreme time consumption. Based on our experiments, MUSCLE performs best with small datasets, but it cannot properly allocate memory resource, resulting in high memory occupancy rate. Hence, MUSCLE eventually reports an out-of-memory message with ultra-large datasets. For large datasets, Mafft and Clustal-Omega are faster than default Muscle and fast Muscle. And Mafft is always the fastest one than Muscle and Clustal-Omega. However, MUSCLE, MAFFT and Clustal-Omega cannot deal with the present ultra-large datasets. Based on Hadoop framework, HAlign and HPTree perform better, but many key-value pair conversion operators also result in high memory occupancy rate. Considering the problems leading to degraded performance, HAlign-II utilizes memory operation on hard disks, cutting down space complexity and memory occupancy rate. These improvements facilitate running of sequence analysis on clusters comprising cheap large-scale and low-end machines. However, HAlign-II features an average SP score that is inferior to those of other methods. Our method ignores high precision for changing large-scale computing power, which is necessary for several decision research.

**Table 2:** Running time and average SPS with genome MSA

	Φ_*DNA*_(1 ×)			Φ_*DNA*_(100 ×)			Φ_*DNA*_(1000 ×)		
	Time	Memory	Avg SPS	Time	Memory (GB)	Avg SPS	Time	Memory (GB)	Avg SPS
MUSCLE	45 m 23 s	~ 8 GB	0.951	–	–	–	–	–	–
MAFFT	1 m 20 s	~ 100 MB	0.926	13 m 21 s	~ 8	0.926	–	–	–
Clustal-Omega	1 h 25 m	~ 3 GB	0.913	15 h 56 m	~ 30	0.913	–	–	–
HAlign	2 m 12 s	~ 300 MB	0.722	26 m 35 s	~ 8	0.722	5 h 28 m	~ 40	0.722
HAlign-II	14 s	~ 100 MB	0.723	10 m 24 s	~ 2	0.723	1 h 25 m	~ 15	0.723

Memory is the maximum memory usage (the same as below)

**Table 3:** Running time and average SPS with RNA MSA

	Φ_*RNA*_(small)			Φ_*RNA*_(large)		
	Time	Memory (GB)	Avg SPS	Time	Memory (GB)	Avg SPS
MUSCLE	1 h 25 m	~ 13	0.821	–	–	–
MAFFT	45 m 33 s	~ 10	0.815	–	–	–
Clustal-Omega	4 h 16 m	~ 10	0.835	–	–	–
HAlign	1 h 32 s	~ 2	0.631	3 h 15 m	~ 10	0.631
HAlign-II	23 m 34 s	~ 1	0.633	59 m 42 s	~ 2	0.633

**Table 4:** Running time and average SPS with protein MSA

	Φ_*Protein*_(1 ×)			Φ_*Protein*_(100 ×)			Φ_*Protein*_(1000 ×)		
	Time	Memory (MB)	Avg SPS	Time	Memory (GB)	Avg SPS	Time	Memory (GB)	Avg SPS
MUSCLE	3 m 13 s	~ 300	0.892	~ 34 h	~ 10	0.892	–	–	–
MAFFT	5 m 53 s	~ 100	0.878	37 m 51 s	~ 5	0.878	7 h 22 m	~ 30	0.878
Clustal-Omega	54 m 08 s	~ 100	0.912	28 h 15 m	~ 5	0.912	–	–	–
SparkSW	3 m 23 s	~ 100	0.716	1 h 12 m	~ 2	0.716	5 h 30 m	~ 18	0.716
HAlign-II	1 m 45 s	~ 100	0.695	26 m 36 s	~ 1	0.695	2 h 52 m	~ 10	0.695

Table 5 presents running times of several outstanding tools on phylogenetic trees construction. IQ-TREE with multiple threads consumes more time than HPTree and HAlign-II, as distributed computing on a single node utilizes multiple threads and features time-efficient data construction. Phylogenetic tree performance is evaluated by maximum likelihood value under log functions. HPTree point reaches – 219,543,85, which is similar to that of NJ model in MEGA [40], implying close performance of results of both methods. Similarly, out-of-memory error occurs when running the HPTree method. Currently, no outstanding method exists for constructing large-scale evolutionary trees, even on workstation clusters. Constructing phylogenetic trees based on MSA results can speed up construction speed.

**Table 5:** Running time during phylogenetic trees construction

	IQ-TREE		HPTree		HAlign-II	
	Time	Memory	Time	Memory	Time	Memory
Φ_*DNA*_(1 ×)	9 m 52 s	~ 100 MB	1 m 25 s	~ 300 MB	27 s	~ 100 MB
Φ_*DNA*_(100 ×)	1 h 2 m	~ 5 GB	45 m 32 s	~ 8 GB	17 m 45 s	~ 1 GB
Φ_*DNA*_(1000 ×)	–	–	–	–	1 h 45 m	~ 10 GB
Φ_*RNA*_(small)	–	–	6 h 23 m	~ 2 GB	52 m 39 s	~ 1 GB
Φ_*RNA*_(large)	–	–	28 h 36 m	~ 10 GB	8 h 20 m	~ 2 GB
Φ_*Protein*_(1 ×)	22 m 12 s	~ 100 MB	Not supported	Not supported	2 m 24 s	~ 100 MB
Φ_*Protein*_(100 ×)	5 h 05 m	~ 5 GB	Not supported	Not supported	33 m 32 s	~ 1 GB
Φ_*Protein*_(1000 ×)	38 h 52 m	~ 30 GB	Not supported	Not supported	3 h 36 m	~ 10 GB

### Memory efficiency and scalability

**Fig. 5:**
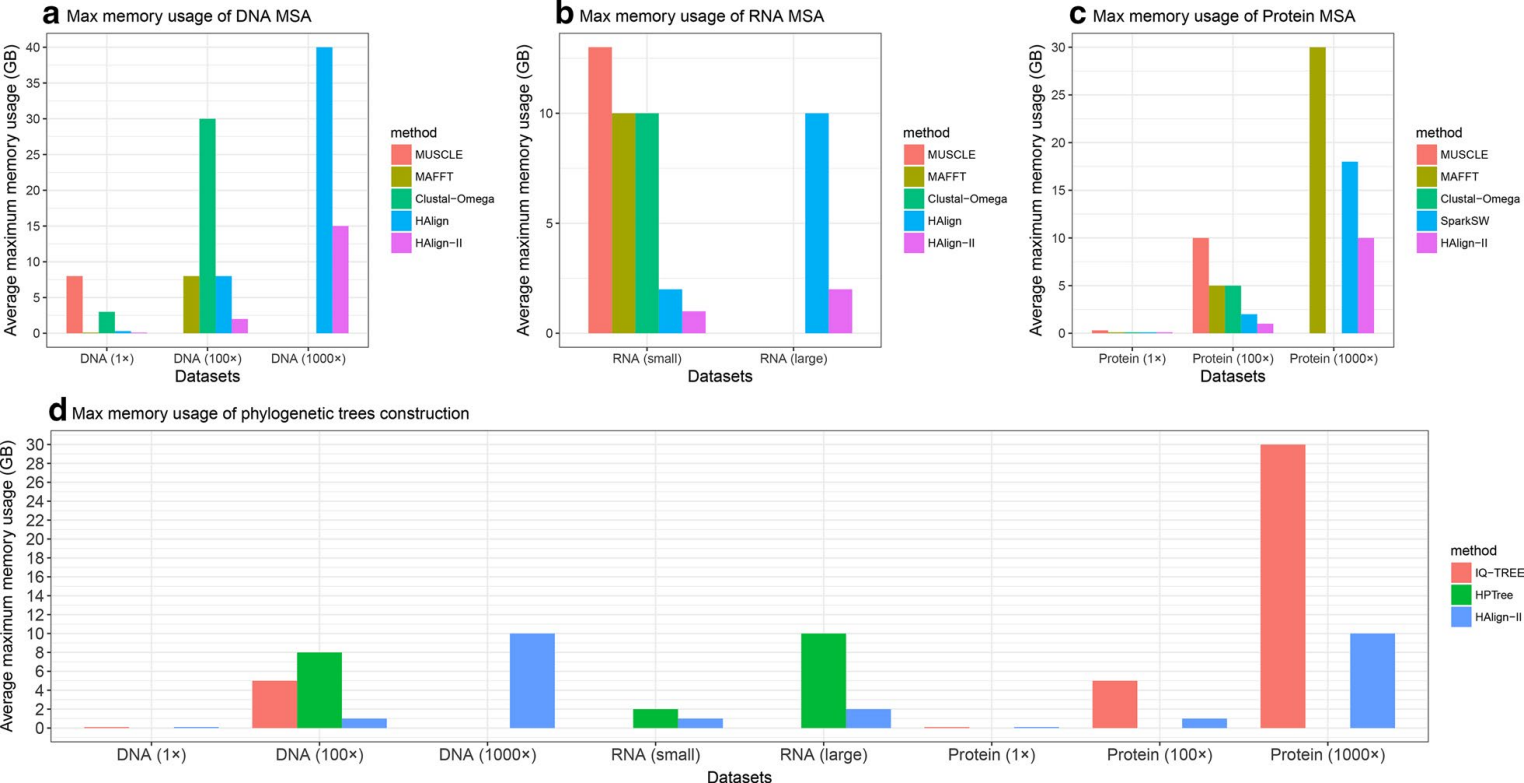
Average maximum memory usage of various experiments

Currently, most time-efficient methods, such as MUSCLE with small datasets and Mafft/Clustal-Omega/HAlign for large-scale datasets, present extremely large space complexities, resulting in impossibility to actually address ultra-large datasets. Based on all experimental results of Tables 2, 3, 4 and 5, we compare memory usage of HAlign-II with other state-of-the-art methods in Fig. 5.

Figure 5 shows average maximum memory usage of each machine on the cluster containing 12 machines for SparkSW/HAlign/HAlign-II and a standalone machine for MUSCLE/Mafft/Clustal-Omega. To conclude, Spark framework exhibits more efficient memory than Hadoop framework, as shown by inferiority of HAlign compared with other methods. Whether for nucleotide sequences or protein sequences, HAlign-II presents the lowest average maximum memory usage, thereby facilitating ultra-large MSA and phylogenetic tree construction on cheaper clusters.

**Fig. 6:**
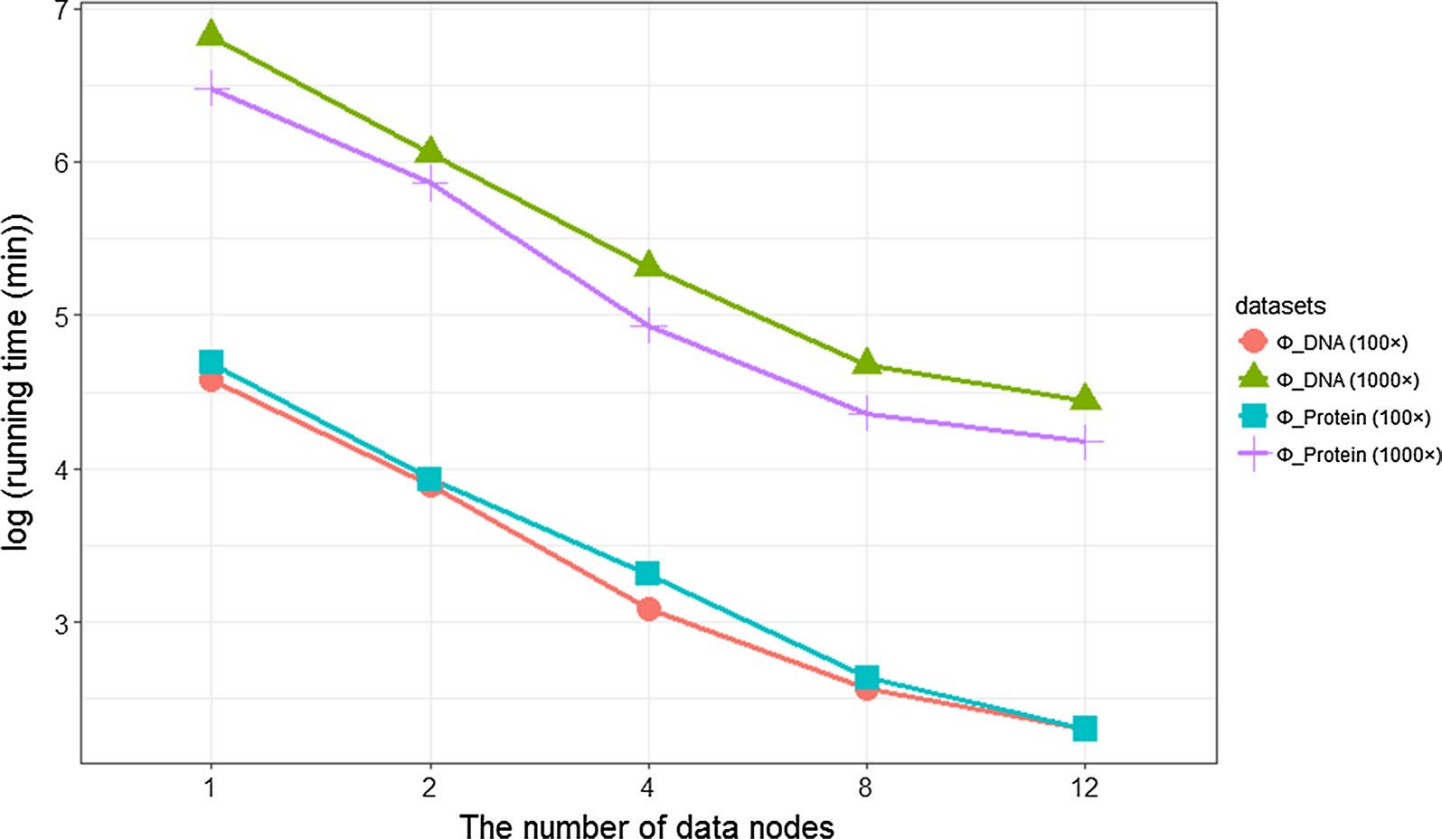
Running time with increasing worker nodes

Additionally, Fig. 6 shows that with increase in worker nodes, running time and memory efficiency becomes significantly low, indicating linear growth of capacity and computing power with increase of such nodes.

## Discussion

Multiple biological sequence alignment and phylogenetic tree construction present complicated inter-relationships, and both are necessary for sequence analysis. In the last several decades, many state-of-the-art methods and algorithms were created for more time- and space-efficient MSA and phylogenetic trees construction issues. With increasing next-generation sequence database, addressing ultra-large datasets became an unprecedented challenge. Other outstanding methods were developed to improve time efficiency even with precision loss; such methods include ClustalW-MPI, Hadoop-BAM, HAlign, and HPTree. Thus, with the urgent need for additional time-efficient and computing power for ultra-large datasets, we conduct a series of experiments to assess the performance of our HAlign-II method.

Based on Spark distributed and parallel computing model, Smith–Waterman algorithm, trie trees, and NJ methods are employed to completely utilize hardware resources and computing power. For ultra-large genome and RNA MSA experiments, MUSCLE, MAFFT and Clustal-Omega achieve high accuracies. However, both traditional tools show complete incompatibility with large datasets. Methods based on distributed computing model present remarkable advantages, especially HAlign-II, which presents the highest memory efficiency. SparkSW and HAlign-II work well for ultra-large protein MSA experiments. However, the former still needs to further cut down memory occupation. Difficulty also arises from insufficient phylogenetic tree construction for ultra-large protein sequences. For ultra-large phylogenetic tree construction based on MSA results, most tools run out of memory, and even nearly 400 GB memory cannot address the requirement of 10 GB size datasets. All experimental results indicate that with regard to ultra-large nucleotide MSA or protein MSA and phylogenetic tree construction, HAlign-II performs best with regard to time efficiency, memory efficiency, and scalability.

## Conclusions

This paper presents a distributed and parallel computing tool named HAlign-II to address ultra-large multiple biological sequence alignment and phylogenetic tree construction. After comparing this tool with a series of state-of-the-art methods with ultra-large data, we conclude that HAlign-II features three advantages: (1) extremely high memory efficiency and good scaling with increases in computing resource; (2) efficient construction of phylogenetic trees with ultra-large biological sequences; (3) provision of user-friendly web server based on high performance and distributed computing infrastructure; the server is established at http://lab.malab.cn/soft/halign. These improvements will be significant in coping with extreme increases in next-generation sequencing.
